# Verb fluency task in individuals with mild cognitive impairment and dementia: a scoping review

**DOI:** 10.1590/1980-5764-DN-2026-0476

**Published:** 2026-08-24

**Authors:** Emily Viega Alves, Fernanda Mambrini Só e Silva, Victória Tizeli Souza, Maira Rozenfeld Olchik, Bárbara Costa Beber, Raphael Machado Castilhos

**Affiliations:** 1 Universidade Federal do Rio Grande do Sul, Programa de Pós Graduação em Ciências Médicas, Porto Alegre RS, Brazil.; 2 Universidade Federal do Rio Grande do Sul, Porto Alegre RS, Brazil.; 3 Universidade Federal de Ciências da Saúde de Porto Alegre, Departamento de Fonoaudiologia, Porto Alegre RS, Brazil.

**Keywords:** Cognitive Dysfunction, Dementia, Verbal Behavior, Neuropsychological Tests, Disfunção Cognitiva, Demência, Comportamento Verbal, Testes Neuropsicológicos

## Abstract

Verb fluency tasks have shown promise in detecting and monitoring neurocognitive disorders, such as mild cognitive impairment (MCI) and dementia, due to their higher syntactic and executive demands.

**Objective:**

To map evidence on the assessment of verb fluency in individuals with MCI and dementia, focusing on methodologies, performance patterns, and diagnostic accuracy.

**Methods:**

A scoping review was conducted following PRISMA-ScR guidelines. Studies assessing verb fluency in individuals with MCI or dementia were included, without language or publication date restrictions. Searches were performed in PubMed, LILACS, SciELO, Embase, and Scopus.

**Results:**

Sixteen studies met the inclusion criteria, most with cross-sectional designs and quantitative analyses. Performance followed a consistent gradient, with individuals with dementia showing the greatest impairment, followed by those with MCI and healthy controls. Evidence suggested predictive value for disease progression, contributions to differential diagnosis, and associations with frontotemporal dysfunction. Diagnostic accuracy was moderate.

**Conclusion:**

Verb fluency performance reflects the severity of cognitive impairment and may complement other clinical and neuropsychological measures, despite methodological variability.

## RESUMO.

A fluência de verbos tem mostrado potencial na detecção e monitoramento de transtornos neurocognitivos, como comprometimento cognitivo leve (CCL) e demência, devido às suas maiores demandas sintáticas e executivas.

**Objetivo:**

Mapear as evidências sobre a avaliação da fluência verbal em indivíduos com CCL e demência, com foco em metodologias, padrões de desempenho e acurácia diagnóstica.

**Métodos:**

Foi conduzida uma revisão de escopo seguindo as diretrizes PRISMA-ScR. Foram incluídos estudos que avaliaram a fluência verbal em indivíduos com CCL ou demência, sem restrições de idioma ou data de publicação. As buscas foram realizadas nas bases PubMed, LILACS, SciELO, Embase e Scopus.

**Resultados:**

Dezesseis estudos atenderam aos critérios de inclusão, sendo a maioria com delineamento transversal e análises quantitativas. O desempenho seguiu um gradiente consistente, com indivíduos com demência apresentando maior comprometimento, seguidos por aqueles com CCL e controles saudáveis. As evidências sugeriram valor preditivo para progressão da doença, contribuições para o diagnóstico diferencial e associações com disfunção frontotemporal. A acurácia diagnóstica foi moderada.

**Conclusão:**

O desempenho em tarefas de fluência verbal reflete a gravidade do comprometimento cognitivo e pode complementar outras medidas clínicas e neuropsicológicas, apesar da variabilidade metodológica.

## INTRODUCTION

 Diagnoses such as Mild Cognitive Impairment (MCI) and dementia are related to the process of population aging^1^. A report published in 2018 estimated that around 50 million people live with dementia, and this number is projected to rise to 152 million by 2050, especially in low- and middle-income countries^2^. Neuropsychological tests play an important role in identifying and characterizing the cognitive changes observed in people with these diagnoses and are especially useful for understanding functions such as memory, attention, orientation, language, and executive functions^3^. In neuropsychological assessment, verbal fluency tasks are commonly administered, particularly because they require different cognitive abilities and can quickly and sensitively demonstrate deficits^4^. 

 In the verbal fluency task, individuals are asked to recall as many words as possible, usually within one minute, based on a specific characteristic or criterion^5^. This task assesses multiple cognitive domains in an integrated manner, including attention, vocabulary, the ability to inhibit the processing of other categories, and the performance of a restricted mental search^6^. There are different types of verbal fluency, which vary according to the category chosen: semantic verbal fluency (generation of words from a specific semantic category, such as animals or fruits), phonemic or orthographic verbal fluency (generation of words beginning with a certain letter, with the letters “f,” “a,” and “s” being the most commonly used), verb or action fluency (generation of verbs or words representing “things people can do”), and free verbal fluency (free recall)^5^. Each type of task differently engages cognitive processes and recruits certain brain areas depending on the demands imposed by the criterion used^5^. In addition, individual characteristics, such as educational level, can interfere with test performance^7^. There are different ways to apply the verbal fluency task. The most traditional approach is to analyze performance through the total number of correctly produced words. Furthermore, analysis can be performed using the clustering and switching methodology^8^ or by evaluating incorrect words^9^. 

 The verb fluency task is especially relevant because it can be considered more challenging than other verbal fluency tasks due to its semantic and syntactic complexity^10^. Studies indicate that verb retrieval involves processes related to the motor aspects of language production and, for this reason, individuals with pathologies involving not only the temporal lobe but also the frontal lobe, which is responsible for coordinating the motor aspects of speech, perform worse^11,12^. Verb fluency has been suggested as a marker of fronto-striatal impairment and a measure of executive and linguistic functioning^13^. 

 Studies investigating the verb fluency task in individuals with Alzheimer disease (AD) have indicated poorer performance compared to their healthy peers^14^. Other studies have also suggested that this test may indicate the conversion of cognitively healthy individuals to MCI^15^. Effectively understanding the relevance of this task in these conditions may be valuable for facilitating early diagnosis and identifying specific associated performance patterns, which could contribute to a better understanding of the mechanisms underlying the observed cognitive deficit. 

 This study aimed to conduct a scoping review to systematically map the findings of studies investigating the assessment of the verb fluency task in individuals with MCI and dementia. Specifically, the review examined the methodologies used for evaluation, the patterns produced by the target population, and the ability of the task to distinguish MCI from dementia. The research question was developed using the population, concept, context (PCC) framework: “What is currently known in the literature about the use of the verb fluency task in individuals with MCI and dementia?” 

## METHODS

 This study adopted a scoping review methodology, which differs from systematic reviews by drawing on a broader spectrum of study designs. Rather than focusing on a narrowly defined question, it aimed to provide an overview of the existing evidence and identify gaps in the literature across diverse research approaches. 

### Protocol and registration

 Our protocol was drafted using the PRISMA Extension for Scoping Reviews (PRISMA-ScR)^16^ and was revised by the research team. After consensus among the authors, the final protocol was registered on the Open Science Framework at https://osf.io/u83qs. 

### Eligibility criteria

 To be included in this review, papers had to assess verb fluency tasks in individuals with MCI or dementia. There were no restrictions regarding publication period or language. Only original research papers were considered. Studies that did not assess verb fluency, that assessed only other types of verbal fluency (such as semantic or phonological fluency), or that did not include individuals with MCI or dementia as the main population were excluded. 

### Information sources

 To identify potentially relevant documents, the following bibliographic databases were searched: Library of Medicine (PubMed), *Literatura Latino-Americana em Ciências da Saúde* (LILACS), Scientific Electronic Library Online (SciELO), Embase, and Scopus. Other sources, such as the references lists of papers included in the review, were also analyzed to identify additional studies. 

### Search

 The search strategies were drafted by an experienced librarian and further refined through team discussion. The terms used were controlled by the Medical Subject Headings (MeSH) vocabulary. The final search strategies used in each database are described in Supplementary Material Table S1 (available at https://www.demneuropsy.org/wp-content/uploads/2026/05/DN-2026.0476-Supplementary-Material.docx). The final search results were exported into Rayyan, and duplicates were removed using the same platform. 

### Selection of sources of evidence

 The first stage of evidence selection was conducted by two reviewers who screened all publications retrieved from each database. Duplicate records were removed, and titles and abstracts of potentially relevant studies were assessed for eligibility. Disagreements in study selection or data extraction were resolved by consensus. 

### Data charting process

 The authors developed a data extraction form to determine which variables would be extracted. The form was created using Microsoft Excel. 

### Data items

 General characteristics of the papers (authors, year, country, and study design), sample characteristics (sample size, age, gender), information regarding the methodology used in the verb fluency task (task duration and type of analysis), as well as patterns produced by the analyzed population and study conclusions, were extracted. 

## RESULTS

**Figure 1:**
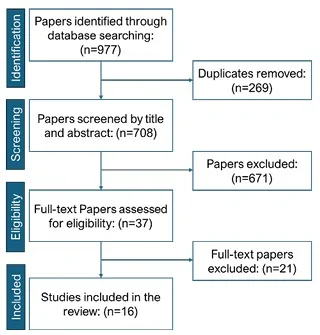
Flowchart of the study selection process. Source: PRISMA.

 After duplicates were removed, 708 publications were identified through searches of electronic databases and references from review articles. Based on the title and the abstract, 671 were excluded, leaving 37 full-text articles to be retrieved and assessed for eligibility. The remaining 37 studies were evaluated in full, resulting in the exclusion of 21 articles: 11 for not addressing the population of interest, 2 for not having undergone peer review, 8 for not assessing verb fluency, and 8 because access to the full text was unavailable (some studies met more than one exclusion criterion). At the end of the process, 16 studies were included in this scoping review. Figure 1 shows the study selection flow in detail. 

 The included studies were published between 1999 and 2023, with a fairly even distribution across the years. Most studies were conducted in the United States (43.75%, n=7), followed by Brazil (18.75%, n=3), France (12.5%, n=2), Sweden (12.5%, n=2), Spain (6.25%, n=1), and Taiwan (6.25%, n=1). The majority of studies employed a cross-sectional design (87.5%), with only one study using a fully longitudinal design and another employing a mixed design (cross-sectional and longitudinal). 

 Among studies including participants with dementia, most (75%) focused on AD. Other dementia types, such as Dementia with Lewy Bodies (DLB) and Primary Progressive Aphasia (PPA), were less frequently examined. Individuals with AD produced between 5 and 11 correct verbs per minute, those with MCI produced between 10 and 14, and cognitively healthy controls produced between 8 and 20. Regarding qualitative measures, participants with AD demonstrated fewer switches and clusters, with switches referring to the ability to shift between different verb subcategories and clusters referring to groups of verbs belonging to the same subcategory. Three studies analyzed neuroimaging data and found that poorer task performance was associated with frontotemporal dysfunction. Longitudinal studies indicated that verb fluency serves as a predictive marker for cognitive decline. Only three studies evaluated the sensitivity and specificity of the task, with sensitivity ranging from 69 to 78% and specificity ranging from 67 to 85%. Detailed study characteristics and main findings are presented in Table 1 ^15,17-31^. 

**Table 1:** Details of the included studies.

Reference	Objective	Sample	VF assessment methodology	Main results	Conclusion
Piatt et al.^17^	To compare lexical, semantic, and action fluency in patients with PD and without dementia.	PD without dementia: mean age=70.3 years (SD=6.64). PD with dementia: mean age=73.9 years (SD=6.36). Controls: mean age=72.86 years (SD=7.51). Total sex distribution: 75 men and 61 women.	Duration: 1 minute Methodology: Analysis of correctly produced verbs	VF was significantly worse in patients with PD and dementia compared with those without dementia and controls. The task was more sensitive in differentiating groups than lexical and semantic fluency.	The VF task may be useful for detecting dementia in the early stages in patients with PD.
Ostberg et al.^18^	To compare noun, verb, and phonological fluency in individuals with varying levels of cognitive impairment.	AD: mean age=72.0 years (SD=10.0). MCI: mean age=66.7 years (SD=10.9). SCD: mean age=66.8 years (SD=11.2).	Duration: 1 minute (6 intervals of 10 seconds) Methodology: Analysis of correctly produced verbs	VF was disproportionately impaired in individuals with MCI, showing a declining pattern over time. Additionally, VF demonstrated greater discriminatory power among the types of fluency evaluated.	Impaired verb fluency may serve as a marker for the diagnosis of MCI.
Ostberg et al.^19^	To investigate the relationship between verb and noun fluency and cerebral perfusion deficits in individuals with different degrees of cognitive impairment.	AD (n=33): mean age=64.8 years (SD=9.4). MCI (n=33): mean age=60.5 years (SD=8.7). SCD (n=33): mean age=56.9 years (SD=7.4).	Duration: 1 minute (6 intervals of 10 seconds) Methodology: Analysis of correctly produced verbs	Individuals with AD produced fewer verbs in the VF task compared with the MCI and SCD groups. Temporal hypoperfusion and lower educational level were predictors of worse performance.	The task differentiated the evaluated groups, with worse performance in AD. Impaired VF may be associated with temporal lobe dysfunction.
McDowd et al.^20^	To understand components of verbal fluency that differentiate groups with AD and PD, focusing on semantic and executive processes.	Young adults: mean age=21.5 years (SD=3.1). Older controls: mean age=72.0 years (SD=5.4). AD: mean age=73.8 years (SD=7.2). PD: mean age=71.9 years (SD=6.0).	Duration: Traditional format: 3 minutes; Alternating format: 6 minutes Methodology: Analysis of correctly produced verbs, perseverations, intrusions, clusters (number and size), switches, response time, and comparison between the two formats.	Young adults had better overall performance, while older adults with AD showed more intrusions, perseverations, and worse verbal fluency. Both the AD and PD groups showed fewer clusters, smaller clusters, and fewer switches. However, the PD group performed worse than the AD group. Individuals with AD and PD responded more slowly initially, with AD showing greater difficulty over time. The alternating format increased cognitive demand, resulting in greater impairment in patients, whereas the traditional format benefited all groups.	VF reveals semantic and executive differences between AD and PD, with processing speed having a greater impact on performance, especially in formats requiring cognitive switching.
Lai and Lin^21^	To investigate action-object semantic impairment in Chinesespeaking individuals with and without AD using naming fluency, VF, and picture naming tasks.	AD: mean age=77.55 years (SD=6.79); 7 women and 13 men. Adult controls: mean age=71.75 years (SD=5.93); 10 women and 10 men. Older controls: mean age=50.30 years (SD=4.74); 10 women and 10 men.	Duration: 1 minute Methodology: Analysis of correctly produced verbs	Patients with AD produced significantly fewer verbs compared with both adult and older controls, with age and disease being the main factors affecting task performance.	VF was a predictor of dementia severity in Chinese-speaking individuals.
Delbeuck et al.^22^	To determine whether performance on the VF task has diagnostic value in differentiating dementia due to AD and DLB.	AD: mean age=71.64 years (SD=9.53); 14 men and 20 women. DLB: mean age=69.26 years (SD=10.02); 11 men and 7 women.	Duration: 1 minute Methodology: Analysis of correctly produced verbs	Individuals with AD performed better on VF compared with those with DLB. Noun fluency was also lower in the DLB group.	VF differentiates individuals with AD from those with DLB, with worse performance observed in DLB. Combining VF with noun fluency increases diagnostic accuracy.
Clark et al.^23^	To evaluate noun, verb, and letter fluency in MCI and AD using lexical similarity measures and gray matter analysis of regions of interest.	AD: mean age=74.7 years (SD=7.8); 7 men and 3 women. MCI: mean age=70.7 years (SD=7.4); 18 men and 5 women. Controls: mean age=70.1 years (SD=6.9); 13 men and 12 women.	Not reported	Controls outperformed the MCI and AD groups in verb fluency. Semantic similarity strongly influenced word proximity in the task. VF was associated with activation of the left dorsal frontal cortex and temporal lobes.	Individuals with AD and MCI show deficits in verb fluency, related to dysfunction in frontotemporal regions.
Beber et al.^24^	To investigate the nature of verb impairment in patients with mild and moderate AD by comparing verb fluency and verb naming tasks.	AD: mean age=77.74 years (SD=5.93); 22 women and 13 men. Controls: mean age=73.14 years (SD=4.82); 22 women and 13 men.	Duration: 1 minute Methodology: Analysis of correctly produced verbs	Patients with AD showed deficits in both naming and VF tasks, with greater impairment observed in naming. Naming performance declined with AD severity, while VF remained relatively stable.	Verb deficits in AD are predominantly semantic, with greater impairment in naming than in VF.
Mirandez et al.^25^	To evaluate which verbal fluency task best discriminates between MCI and healthy individuals, analyze whether partial scores improve this discrimination, and investigate the correlation with CSF biomarkers for AD in the MCI group.	MCI: mean age=74.5 years (SD=5.4); 22 women and 8 men. Controls: mean age=72.5 years (SD=4.5); 26 women and 11 men.	Duration: 1 minute Methodology: Analysis of correctly produced verbs	Individuals with MCI produced significantly fewer verbs, especially in the second half of the VF task. There was no significant correlation between performance and CSF biomarkers, possibly due to the small sample size.	VF discriminated between MCI and healthy individuals. However, animal fluency alone and combined with letter fluency was slightly superior in discriminating controls from individuals with MCI.
Alegret et al.^15^	To investigate VF deficits in individuals with MCI and AD, assess the task’s potential for tracking disease progression, and propose normative scores for the Spanish population.	AD: mean age=79.0 years (SD=7.6); 235 women and 132 men. MCI: mean age=71.7 years (SD=8.9); 526 women and 369 men. Controls: mean age=63.3 years (SD=7.8); 391 women and 177 men.	Duration: 1 minute Methodology: Analysis of correctly produced verbs	Verb fluency performance declined significantly from healthy controls to MCI and AD, with lower scores associated with older age and lower educational level. In a longitudinal follow-up (mean duration of 16.6 to 19.3 months), verb fluency predicted conversion from healthy cognition to MCI (18.2%) and from MCI to dementia (21.6%).	VF was a sensitive and predictive marker for conversion from healthy cognitive status to MCI and dementia.
Macoir et al.^26^	To evaluate the contribution of word production, especially verbs compared with nouns, for detecting cognitive impairment.	MCI: mean age=71.05 years (SD=6.1); 9 men and 11 women. SCD: mean age=66.4 years (SD=6.0); 4 men and 16 women. Controls: mean age=70.8 years (SD=7.1); 6 men and 14 women.	Duration: 1 minute Methodology: Analysis of correctly produced verbs, repeated words, errors, clustering and switching analyses (cluster size and number of switches), and word count across three time intervals (1–20s, 21–40s, 41–60s).	Verb production was lower in the MCI and SCD groups compared to controls, with no significant differences in errors or repetitions. Cluster size and number of switches were similar between controls and individuals with SCD. All groups showed a decline in verbal production over time.	VF is sensitive in detecting cognitive impairment in SCD, supporting its role as an early marker in the progression from healthy aging to MCI and dementia.
Paek et al.^27^	To compare brain activation patterns between individuals with dementia and healthy older adults to identify regions associated with VF.	Dementia: mean age=68.11 years (SD=5.78); 2 men and 5 women. Controls: mean age=70.57 years (SD=7.55); 3 men and 6 women.	Duration: 90 seconds (3 intervals of 30 seconds each, with 10-second breaks in between) Methodology: Analysis of correctly produced verbs	Controls produced more verbs in intervals 2 and 3. Patients with dementia showed greater bilateral frontal activation. Medial temporal lobe activation was associated with worse VF performance.	Decline in VF task performance can be used not only as a cognitive marker of dementia but also as a neural marker of frontal and temporal lobe deficits observed in neurodegenerative diseases.
Paek^28^	To examine whether individuals with amnestic AD produce verbs with higher emotional valence in VF responses compared with cognitively healthy individuals.	AD: mean age=78.6 years (SD=8.79); 5 men and 10 women. Controls: mean age=73.0 years (SD=7.08); 7 men and 10 women.	Duration: 30 seconds Methodology: Analysis of correctly produced verbs	Individuals with amnestic AD produced more verbs with higher emotional valence (more positive words) in VF compared with cognitively healthy individuals.	The higher emotional valence observed in individuals with AD may reflect emotional compensation associated with verbal memory impairment.
Scheffel et al.^29^	To verify whether verbal fluency differentiates early variants of PPA and PAS before symptom overlap.	Logopenic PPA: mean age=66.3 years (SD=8.5); 29 women and 28 men. Semantic PPA: mean age=66.8 years (SD=6.7); 9 women and 6 men. Nonfluent PPA: mean age=68.5 years (SD=9.5); 15 women and 19 men. PAS: mean age=70.3 years (SD=9.2); 17 women and 13 men.	Duration: 1 minute Methodology: Analysis of correctly produced verbs	All groups showed abnormal performance on VF, but the task did not distinguish among PPA variants. Individuals with PAS performed better. VF showed a moderate correlation with aphasia severity.	VF does not differentiate among early variants of PPA, but performance is moderately associated with aphasia severity.
Beber et al.^30^	To investigate VF performance in individuals with AD and healthy older adults, focusing on measures such as total words, clusters, and switches.	AD: mean age=78.6 years (SD=6.7); 20 women and 9 men. Controls: mean age=71.5 years (SD=6.3); 34 women and 5 men.	Duration: 1 minute Methodology: Analysis of correctly produced verbs, clustering and switching analyses (number of clusters, cluster size, and number of switches)	Individuals with AD produced fewer switches and fewer correct verbs compared to healthy older adults. There were no significant differences in the number or size of clusters between groups.	VF performance in AD predominantly reflects executive deficits, as evidenced by the reduced number of switches.
Fisher et al.^31^	To investigate the reliability of subjective clustering methods and the feasibility of an objective clustering method in the analysis of VF in AD and controls.	AD: mean age=78.15 years (SD=8.02); 15 women and 5 men. Controls: mean age=72.27 years (SD=6.94); 14 women and 8 men.	Duration: 1 minute (if the participant understands the command immediately) or 30 seconds (if there are two incorrect responses or initial non-comprehension). Methodology: Analysis of clusters and switches (total number of words, number of clusters, cluster size, and number of switches), combining subjective and objective analyses based on the Lancaster Norms database.	Individuals with AD produced significantly fewer words, clusters, and switches than controls, regardless of method (subjective or objective). Cluster size did not differ significantly between groups.	Objective clustering methods, such as those based on the Lancaster Norms, are feasible for analyzing VF in AD, showing agreement with subjective methods. Subjective analysis may help reduce bias and increase replicability.

Abbreviations: VF, Verb Fluency; PD, Parkinson’s Disease; SD, Standard Deviation; AD, Alzheimer Disease; MCI, Mild Cognitive Impairment; SCD, Subjective Cognitive Decline; DLB, Dementia with Lewy Bodies; CSF, Cerebrospinal Fluid; PPA, Primary Progressive Aphasia; PAS, Progressive Apraxia of Speech.

Source: The authors.

## DISCUSSION

 Verbal fluency tests, such as semantic and phonological fluency, are well-established tools in the diagnosis of neurocognitive disorders^32^. However, the verb fluency task is less frequently mentioned, even in neuropsychological assessment batteries^33-35^, likely due to the limited number of studies that have thoroughly explored its clinical relevance. This scoping review aimed to systematically map and synthesize findings from studies investigating the use of the verb fluency task in two diagnoses: MCI and dementia. Through this work, the available evidence regarding the use of the verb fluency task was mapped, including reported diagnostic accuracy, its potential to distinguish between conditions, the methodologies used, and possible cognitive and neural correlates. 

 In general, the findings of this review indicate that verb fluency is a sensitive and potentially predictive task for the identification and progression of neurocognitive conditions, such as MCI and dementia, although these findings should be interpreted with caution because of the limited number of longitudinal studies. Alegret et al.^15^ demonstrated that verb fluency performance predicted both the conversion from healthy cognition to MCI and from MCI to dementia, emphasizing its longitudinal clinical utility. Additionally, studies such as Delbeuck et al.^22^ and Piatt et al.^17^ highlighted the value of verb fluency in differential diagnosis, successfully distinguishing between conditions such as AD and DLB, or between patients with Parkinson’s disease with and without dementia. However, this discriminative power does not appear to extend to differentiating subtypes within the same clinical entity. As shown by Scheffel et al.^29^, verb fluency was not effective in distinguishing between early variants of primary progressive aphasia, despite overall impaired performance. Despite these findings, few studies have addressed the accuracy of the test, and the available data indicate that both sensitivity and specificity are only moderate, which may limit its diagnostic utility. Based on this, it is clear that the verb fluency task should not be used alone but rather as part of a broader set of neuropsychological, clinical, and laboratory assessments. Its combined use may increase diagnostic accuracy, improve monitoring of disease progression, and provide a more complete picture of patient’s cognitive functioning. 

 Our study found heterogeneity in the objectives and methodologies of the included studies. This variability is expected in scoping reviews, which are designed to map the extent and nature of research on a topic rather than synthesize results quantitatively^36^. In this review, this heterogeneity was reflected in some key aspects. First, the objectives varied widely: while some studies aimed to evaluate verb fluency as a diagnostic tool or predictor of disease progression, others focused on comparing the test with naming tasks or examining correlations with neuroimaging and biomarkers. There was also variability in time limits: although most studies used a one-minute verb fluency task, variations were observed in instructions, duration (30 seconds to 3 minutes), and response format (*e.g.*, traditional *vs.* alternating). Another important point is that the assessment methods ranged from counting only correct verbs to more complex analyses involving error types, response time, clustering, switching, and even emotional valence. Greater standardization in the administration and reporting of verb fluency tasks would be important as a future direction. 

 Studies using neuroimaging^19,23,27^ indicate that verb fluency is associated with the integrity and activation of frontotemporal regions, especially the frontal and medial temporal lobes. These findings suggest that the task may reflect brain changes in neurodegenerative diseases. The only study that evaluated the correlation between verbal fluency performance (including the verb fluency task) and biochemical biomarkers in cerebrospinal fluid^25^ did not find a significant association, highlighting the need for further investigation with larger samples. 

 The vast majority of studies evaluated the task quantitatively by counting the number of correct verbs, possibly because it is an objective, standardizable measure that is simple to apply clinically and in research. Studies that also included clustering and switching analyses in their methodology offered a more refined qualitative assessment of the cognitive processes underlying the task. Studies such as Beber et al.^30^ and Fisher et al.^31^ showed that individuals with dementia tend to switch less, suggesting executive difficulties, even when the total number of verbs is not drastically reduced. Macoir et al.^26^ and McDowd et al.^20^ explored how cluster size and switching frequency behave over time or across different diagnostic groups, contributing to the discrimination between stages of cognitive impairment. Although less commonly addressed, some authors^20,26^ included analyses of errors in verb fluency, such as repetitions, intrusions, and perseverations. The analysis of these characteristics can also add an important qualitative dimension, as they may reflect inhibitory control deficits, often linked to executive dysfunction, as well as a decline in semantic specificity, meaning a tendency to use more general or vague terms instead of precise verbs, indicating degradation in the organization of or access to semantic memory^37^. Moreover, analyzing error patterns complements overall performance measures, offering a more comprehensive understanding of language and cognitive impairments. 

 This scoping review has some limitations. The number of longitudinal studies was small, limiting the ability to draw robust conclusions about the predictive value of verb fluency performance over time or its role in tracking disease progression. The limited number of countries represented in the studies, as well as the high-income status of most of them, also highlights the lack of socioeconomic, cultural, and educational variation, limiting the generalizability of the findings. There was also a lack of studies focusing on less common forms of dementia and on the qualitative aspects of verb fluency tasks. Additionally, in line with the methodology of scoping reviews, no formal methodological quality or risk-ofbias assessment of the included studies was conducted. Therefore, findings related to diagnostic performance and predictive value should be interpreted with caution. These gaps underscore the need for future studies to include more diverse clinical populations, expand the number of longitudinal studies, and explore not only quantitative outcomes. 

 In conclusion, the findings indicate that verb fluency is a sensitive task for identifying cognitive deficits and has the potential to distinguish between stages of impairment, although its diagnostic accuracy is still moderate and depends on combination with other clinical and neuropsychological measures. This study also revealed that, despite methodological differences and varying objectives among the included studies, specific performance patterns, such as reductions in total verb production and changes in the use of cognitive strategies, are observed in the assessed population through the task. Thus, the task shows promise but requires standardization and further investigation, especially in more diverse samples, longitudinal studies, and studies using qualitative approaches, in order to strengthen its clinical applicability in cases of MCI and dementia. 

## Data Availability

No new data were generated or analyzed in this study.
