# Supplementary material for: Verb fluency task in individuals with mild cognitive impairment and dementia: a scoping review
Source: Dement Neuropsychol. 2026 Aug 24;20:e20260476. doi: 10.1590/1980-5764-DN-2026-0476 (PMC13506176; doi:10.1590/1980-5764-DN-2026-0476)
Supplement: Supplementary file 1 [file 1980-5764-dn-20-e20260476-md01.docx]

**Supplementary Material**

**Table S1.** Database search strategies

| Database | Search strategy |
| --- | --- |
| PubMed | (verb fluency[tiab] OR action fluency[tiab] OR verb[tiab]) AND (cognitive dysfunction[mh] OR cognitive decline[tiab] OR cognitive disorder[tiab] OR cognitive impairments[tiab] OR mild cognitive impairment[tiab] OR dementia[mh] OR alzheimer dementia[tiab] OR frontotemporal lobar degeneration[tiab] OR mixed dementia[tiab] OR lewy body dementia[tiab] OR vascular dementia[tiab] OR primary progressive aphasia[tiab] OR parkinson disease[mh]) |
| LILACS | (verb fluency OR action fluency OR verb) AND (cognitive dysfunction OR cognitive decline OR cognitive disorder OR cognitive impairments OR mild cognitive impairment OR dementia OR alzheimer dementia OR frontotemporal lobar degeneration OR mixed dementia OR lewy body dementia OR vascular dementia OR primary progressive aphasia OR parkinson disease) |
| Scielo | (verb fluency OR action fluency OR verb) AND (cognitive dysfunction OR cognitive decline OR cognitive disorder OR cognitive impairments OR mild cognitive impairment OR dementia OR alzheimer dementia OR frontotemporal lobar degeneration OR mixed dementia OR lewy body dementia OR vascular dementia OR primary progressive aphasia OR parkinson disease) |
| Embase | ('verb fluency':ti,ab OR 'action fluency':ti,ab OR 'verb':ti,ab) AND ('cognitive dysfunction'/exp OR 'cognitive decline':ti,ab OR 'cognitive disorder':ti,ab OR 'cognitive impairments':ti,ab OR 'mild cognitive impairment':ti,ab OR 'dementia'/exp OR 'alzheimer dementia':ti,ab OR 'frontotemporal lobar degeneration':ti,ab OR 'mixed dementia':ti,ab OR 'lewy body dementia':ti,ab OR 'vascular dementia':ti,ab OR 'primary progressive aphasia':ti,ab OR 'parkinson disease'/exp) |
| Scopus | (TITLE-ABS("verb fluency") OR TITLE-ABS("action fluency") OR TITLE-ABS("verb")) AND (TITLE-ABS("cognitive dysfunction") OR TITLE-ABS("cognitive decline") OR TITLE-ABS("cognitive disorder") OR TITLE-ABS("cognitive impairments") OR TITLE-ABS("mild cognitive impairment") OR TITLE-ABS("dementia") OR TITLE-ABS("alzheimer dementia") OR TITLE-ABS("frontotemporal lobar degeneration") OR TITLE-ABS("mixed dementia") OR TITLE-ABS("lewy body dementia") OR TITLE-ABS("vascular dementia") OR TITLE-ABS("primary progressive aphasia") OR TITLE-ABS("parkinson disease")) |
